# Follow-Up Analysis of Pulmonary Function, Exercise Capacity, Radiological Changes, and Quality of Life Two Months after Recovery from SARS-CoV-2 Pneumonia

**DOI:** 10.3390/medicina57060568

**Published:** 2021-06-03

**Authors:** Edita Strumiliene, Ingrida Zeleckiene, Rytis Bliudzius, Arturas Samuilis, Tadas Zvirblis, Birute Zablockiene, Arunas Strumila, Vygantas Gruslys, Laura Malinauskiene, Vytautas Kasiulevicius, Ligita Jancoriene

**Affiliations:** 1 Department of Pulmonology and Allergology, Vilnius University Hospital Santaros Klinikos, Santariskiu 2, LT-08661 Vilnius, Lithuania; vygantas.gruslys@santa.lt (V.G.); laura.malinauskiene@santa.lt (L.M.); 2 Centre of Radiology and Nuclear Medicine, Vilnius University Hospital Santaros Klinikos, Santariskiu 2, LT-08661 Vilnius, Lithuania; Ingrida.zeleckiene@santa.lt (I.Z.); rytis.bliudzius@santa.lt (R.B.); arturas.samuilis@santa.lt (A.S.); 3 Faculty of Medicine, Vilnius University, M.K. Ciurlionio 21/27, LT-03101 Vilnius, Lithuania; birute.zablockiene@santa.lt (B.Z.); arunas.strumila@santa.lt (A.S.); Vytautas.Kasiulevicius@mf.vu.lt (V.K.); ligita.jancoriene@santa.lt (L.J.); 4 Institute of Mechanical Science, Vilnius Gediminas Technical University, Sauletekio al. 11, LT-10223 Vilnius, Lithuania; tadas.zvirblis@vilniustech.lt; 5 Center of Infectious Diseases, Vilnius University Hospital Santaros Klinikos, Santariskiu 2, LT-08661 Vilnius, Lithuania; 6 Department of Pediatric Surgery, Vilnius University Hospital Santaros Klinikos, Santariskiu 2, LT-08661 Vilnius, Lithuania

**Keywords:** COVID-19, pneumonia, follow-up, consequences

## Abstract

*Background and objective*: According to the World Health Organization (WHO), more than 100 million people have already recovered from SARS-CoV-2 infection. Therefore, it is imperative to understand the possible outcomes of COVID-19. The aim of our study was to evaluate pulmonary function, exercise capacity, residual radiological changes, and health-related quality of life (HRQoL) at follow-up in a cohort of SARS-CoV-2 pneumonia survivors. *Materials and Methods:* Patients with SARS-CoV-2 infection and radiologically confirmed lung injury, with no chronic lung disease prior to this infection, were included in the study. Patients’ evaluation 2 months after their discharge from hospital included spirometry (FVC, FEV1, FEV1/FVC), determination of lung volume (TLC, VC, RV) and diffusing capacity of lung for carbon monoxide (DLCO, adjusted for hemoglobin), 6-Minute Walk Test (6MWT), chest CT scan, and 36-Item Short Form General Health Survey (SF-36). *Results:* Fifty-one patients (25 men, 26 women) were included. The mean age was 56 years (SD-11,72). Eighteen patients (35.3%) had experienced moderate COVID-19, 21 (41.2%) severe COVID-19, and 12 (23.5%) were critically ill. The mean follow-up visit time after the discharge from hospital was 60 days (SD-17). Pulmonary function at follow-up was impaired in 24 (47.2%) patients. Reduced lung volume was observed in 15 (29.4%) patients, DLCO reduction in 15 (29.4%) patients, and only one patient displayed obstruction. Twelve patients out of 51 (12/51, 27.3%) showed reduced physical capacity in the 6 MWT, and 3/51 (9.1%) showed desaturation, with SO2 < 90%. Different levels of abnormality were found in 49/51 (96,1%) patients on follow-up chest CT; the median radiological score was 10.9 (SD ± 8.87, possible maximal score, 25). Ground-glass opacity was the most common radiological feature, found in 45 (88.2%) patients. The SF-36 scores demonstrated a reduction in health status across all domains, with the lowest scores for limitations in social activities because of physical problems, vitality, and general health. *Conclusion:* In the group of COVID-19 pneumonia survivors 2 months after hospital discharge, residual changes in the lungs on chest CT and in lung function and reduced physical and HRQoL status were found in a significant number of patients. To evaluate COVID-19 long-term consequences, a longer follow-up period is needed.

## 1. Introduction

Coronavirus disease 2019 (COVID-19), caused by the severe acute respiratory syndrome coronavirus-2 (SARS-CoV-2), was first diagnosed in December 2019 in Wuhan, China [1]. The disease spread rapidly over the world, and more than 73 million people have been infected during one year. According to the World Health Organization (WHO), SARS-CoV-2 was the cause of death of 1.5 million people, but more than 60 million have survived [2]. In this situation, it is imperative to understand how COVID-19 impacts on patients’ physical and mental status as well as quality of life, the duration of the recovery period, and the possible short-term and long-term outcomes of this disease.

According to the WHO, up to 80% of SARS-CoV-2 infections are mild, 15% of the patients develop severe symptoms, and 5% become critically ill [2]. 

Lung injury is one of the most common clinical features of this infection [1,3]. In severe cases, lung injury is often presenting as bilateral pneumonia with respiratory failure and/or ARDS and is the major cause of mortality in the acute phase [2,4].

The short-term and long-term sequelae of lung injury in COVID-19 survivors is not well known yet. The data of a few recent studies show that changes in the lungs on CT scans and impaired lung function are still observed 4–6 or even 12 weeks after the onset of the symptoms [5,6], but improve over time [7].

Survivors of genetically similar severe acute respiratory virus (SARS) infections showed a mild or moderate restrictive impairment of lung function 6–8 weeks after hospital discharge, with muscle weakness in 6–20% of subjects [8]. Residual fibrotic lung changes were observed in up to 20% of patients after SARS [9]; moreover, lung fibrosis was observed in 1/3 of patients after Middle East respiratory syndrome (MERS) infection [10].

The aim of our study was to evaluate and describe pulmonary function, exercise capacity, radiological changes, and health-related quality of life at follow-up in a cohort of patients who recovered from SARS-CoV-2 pneumonia.

## 2. Materials and Methods

### 2.1. Patients

Consecutive eligible adult patients with a real-time reverse transcriptase polymerase chain-reaction (RT-PCR) test-confirmed SARS-CoV-2 infection, treated in the Vilnius University Santaros Klinikos tertiary hospital from 1 May 2020 to 1 October 2020 were offered the standard pulmonological follow-up at the respiratory outpatient department 2 months after their discharge. Patients were included in the study if they matched these inclusion criteria:

- radiologically chest X-ray- and/or CT-confirmed lung injury
- they were willing and capable to complete the pulmonary functions test, 6-min walking test, and the health status -elated quality of life short form questionnaire (SF-36) at follow-up
- no chronic lung disease prior the infection (to avoid radiological and functional overdiagnosis)
- they were able to understand and sign the informed consent to participate in the study.

Patients with mild COVID-19 were not included in the study, as they did not have lung injury, according to the classification of WHO and Chinese Clinical Guidance [11,12].

The patients with COVID-19, included in the study, were categorized into the following groups:

- moderate disease (clinically and radiologically confirmed pneumonia, with no requirement for supplemental oxygen; oxygen saturation >93%)
- severe disease (having radiological evidence of bilateral pneumonia with lung injury >v50% and any of the following: respiratory rate ≥30 breaths/min, oxygen saturation ≤93% at rest; oxygen therapy ≤10 L/min; no need for treatment in the Intensive Care Unit (ICU));
- critical disease (respiratory failure with the need of high-flow oxygen therapy or intubation, shock, or other organ failure that requires ICU care).

### 2.2. Data Collection

From the hospital database, the medical records of the participants were reviewed, and the demographic data, comorbidities, clinical and laboratory tests results, and chest radiological data at the time of hospitalization (acute disease) were analyzed.

At the control visit 2 months after the discharge from the hospital, chest a CT scan, lung function tests, and the 6-Minute Walk Test (6 MWT) were performed; patients completed 36-SF questionnaire.

#### 2.2.1. Pulmonary Function Testing and 6 MWT

The pulmonary function tests were performed using Vmax Encore (Viasys^®^ Healthcare, USA). The following parameters were measured: forced vital capacity (FVC), forced expiratory capacity at the first second of exhalation (FEV1), FVC/FEV1 ratio, total lung capacity (TLC), vital capacity (VC), residual volume (RV), diffusion capacity of the lung for carbon monoxide (DLCO) measured by means of the single-breath test. The DLCO was corrected by the hemoglobin value. Pulmonary function tests were analyzed based on the ATS-ERS guidelines [13]. All parameters were expressed as percentages of the predicted normal value; the lower limits of normal (LLN) were taken into account.

The 6-minute Walk Test (6 MWT) was performed according to ATS/ERS guidelines, with baseline SpO2 measured by pulse oximetry [14,15].

#### 2.2.2. Chest CT Protocols and Image Analysis

At the time of admission, all patients with suspected COVID-19 pneumonia underwent posterior–anterior and lateral chest X-rays. In cases in which X-rays were indeterminate or normal and a high clinical suspicion of pneumonia was present, chest CT examinations were performed. The initial X-rays and CT scans were followed-up by CT scans 2 months later for all patients. For the portion of our cohort who did not undergo a CT scan at the initial stage of disease, posterior–anterior and lateral chest X-rays were used for the assessment of disease extent.

All chest CT scans were performed using two helical CT scanners: (1) 64-row GE (General Electric Healthcare, Waukesha, Wisconsin, United States) Discovery CT750 HD. A low-dose lung CT protocol was used. All CT images were acquired at the end of inhalation. Acquisition parameters were set as follows: tube voltage 100 kV, automatic tube current modulation, SmartmA/AutomA, FOV, Large Body, detector coverage, 40 mm, slice thickness 3.75 mm, pitch, 1.375, gantry rotation time, 0.4 s. All images were then reconstructed with a slice thickness of 1.25 mm using lung and standard algorithms. Adaptive Statistical Iterative Reconstruction (ASiR) with 30% mA reduction was used for the reduction of the X-ray dose. (2) BrainLab Airo mobile CT (Manufacturer Mobius Imaging, LLC., Shirley, MA, USA), Chest CT protocol was used. Acquisition parameters were set as follows: tube voltage 120 kV, tube current, 50 mA, rotation time, 1.92 s, FOV, Large Body, detector coverage, 40 mm, slice thickness, 1.25 mm, pitch, 1.415. All images were reconstructed using lung and standard algorithms.

Image analysis was performed by two independent radiologists. For studies where a discordance of scoring was found, further review was performed by the third radiologist until a consensus was reached.

CT features such as ground-glass opacities (GGO), consolidation, parenchymal bands and architectural distortion, air bronchograms, bronchiectasis, as well as their distribution were noted. The findings were classified into two groups as either inflammatory changes, being GGO and consolidation, or fibrotic/reticular changes (parenchymal bands, architectural distortion, bronchiectasis). Involvement of each lung lobe was quantified using a method previously used in other studies for evaluating pulmonary fibrosis caused by SARS. Each of the 5 lung lobes were given a score of 0–5 points for inflammatory and fibrotic/reticular changes. Points were given as follows: 0 for a lobe without perceptible changes, 1 for lesions involving up to 5% of a lobe, 2 for lesions involving 6–25% of a lobe, 3 for lesions involving 26–50% of a lobe, 4 for lesions involving 51–75% of a lobe, 5 for lesions involving more than 75% of a lobe [16,17]. In baseline studies, points of each lobe were added together, with a maximum summed score of 25, and one score was attributed for the extent of lesions, not taking into account the type of CT features; in contrast, in follow-up studies, two scores were attributed for inflammatory and fibrotic changes accordingly, and a total radiological score was determined.

#### 2.2.3. SF-36 Questionnaire

The SF-36 questionnaire consists of 36 question that evaluate 8 health domains: physical functioning (PF), social functioning (SF), role limitation due to physical problems (RP), role limitation due to emotional problems (RE), mental health (MH), bodily pain (BP), vitality (VT), and general health (GH).

Scores for each aspect range from 0 (worst) to 100 (best), with higher scores indicating better health-related quality of life (HRQoL). Standard test-scoring algorithms and related interpretation were used [18]. The test was completed by each patient, and the results were compared between the disease severity groups and with those of the general Dutch populations [19].

#### 2.2.4. Laboratory Parameters

The following laboratory parameters were analyzed during the acute diseases phase: total blood leukocyte and lymphocyte counts and concentration of C-reactive protein, D-dimers, interleukin-6 (IL-6), ferritin lactate dehydrogenase (LDH); quantitative serum creatinine and liver enzymes (AST, asparagine aminotransferase; ALT, alanine aminotransferase) were analyzed to evaluate the injury of kidneys and liver.

### 2.3. Statistical Analysis

Descriptive statistics such as frequency tables and mean (standard deviation) were used to describe quantitative and qualitative data, respectively. Normality of quantitative variables was assessed by the Kolmogorov–Smirnov test. Differences between two independent quantitative and qualitative groups were evaluated by the Student’s t-test and Fisher exact test, respectively. One-way ANOVA was used to evaluate the mean differences when comparing more than 2 groups. Bonferroni correction was used for pairwise comparisons. A two-tailed p-value less than 0.05 was considered to be significant. Statistical analysis was performed using Statistical Analysis System (SAS) package version 9.2.

## 3. Results

### 3.1. Demographic Data

Sixty consecutive eligible patients were offered to participate in the study: 51 patients were prospectively enrolled, 5 refused, and 4 were excluded due to a first-time diagnosed chronic obstructive lung disease (COPD) at the control visit (symptoms, smoking history, and spirometry data suggested the presence of undiagnosed COPD prior to COVID-19).

The mean age of the patients was 56 (SD-11.72) years; 26 (51.0%) patients were female. Most of the patients were in the 51–60 (35.3%) and 41–50 (25.5%) age groups. All patients were Caucasians.

The mean time of the control visit was 60 days (SD-17) after discharge from the hospital.

No active smokers were in the study group, and four (7.8%) patients were ex-smokers (their history of smoking was less than 5 years).

Only three patients (5.9%) had no remaining symptoms at the follow-up visit. The most common residual symptoms were fatigue (68.6%), reduction of physical activity (60.8%), dyspnea at exertion (54.9%), and asthenia (37.3%); less common symptoms were cough, arthralgias, hair loss, insomnia, and headache.

More than half of the patients (54.9%) were obese (mean body mass index (BMI) of 31.17 (SD-5.95)), and half of the patients had comorbidities.

The demographics and clinical characteristics of the patients are summarized in Table 1.

Patients were classified in groups according to disease severity: 18 (35.3%) patients had moderate disease, 21 (41.2%) had severe disease, and 12 (23.5%) were recovering after critical disease (the mean treatment time spent in the intensive care unit (ICU) was 10 days; 1 patient needed invasive ventilation).

The statistical analysis showed that a significantly higher number of patients in the critical disease group were men (*p* < 0.001). Also, a high correlation between II–III-degree obesity or presence of any comorbidity with disease severity (*p* = 0.027 and *p* = 0.004 respectively) was observed. No significant difference between the disease severity groups was found according to residual symptoms at follow-up visit.

### 3.2. Lung Function Testing and 6 MWT

Pulmonary function at follow-up was impaired in 24/51 (47.2%) patients.

Only one patient showed obstructive impairment, but reduced FEV1 was observed in five (9.8%) patients, with a positive correlation with disease severity.

Reduced lung volume was found in 1/3 of patients: TLC reduction was observed in 11/51 (21.6%) patients (mean 97.0%, range 53–104%), and VC reduction in 9/51 patients (17.6%; mean 105.4%, range 60–161%). Reduced DLCO was observed in one out of three of the patients, with median value of 89.0% (range 52–138).

Details of lung function, 6 MWT, and radiological scoring analysis are shown in Table 2.

Residual pulmonary function impairment may be considered complex, as reduction of DLCO showed statistically significant association with the impairment of other lung function parameters, i.e., reduced lung volumes, FVC (*p* = 0.017), TLC (*p* = 0.035), VC (*p* = 0.004), and FEV1 (*p* = 0.012).

The impairment of DLCO also showed significant correlation with comorbidities such as arterial hypertension with cardiac failure (*p* = 0.037) and diabetes (*p* = 0.005) and was associated with a reduction of physical capacity in the 6 MWT (*p* = 0.029).

When analyzing the association between lung function and disease severity, we found differences regarding FVC (*p* = 0.01), VC (*p* = 0.002), TLC (*p* = 0.051), and DLCO (*p* = 0.063) values. The more severe the disease, the greater the impairment detected.

Twelve patients (27.3%) had reduced physical capacity in the 6 MWT. The median value was 101.5% of the optimal value (range 70–131%). Three patients (9.1%) showed desaturation to SO2 < 90% during the 6 MWT. The 6 MWD (6 min walking distance in m) showed a negative correlation with disease severity in absolute numbers as well as in percentage of estimated distance (*p* = 0.047 and *p* = 0.008, respectively).

### 3.3. Radiological Data

At the time of acute disease, bilateral pneumonia was confirmed by chest X-ray in 23 patients; therefore a CT-scan was not performed. All other patients during acute disease and all patients at the follow-up visit underwent chest CT.

At the time of acute disease, abnormalities in the lungs were found in both lungs in all patients, with a mean radiological score of 13.6 (SD-5.77). The distribution of abnormalities was almost similar in both lungs, with predominance in the right lung—the mean right lung (RL) score was 7.7 (SD-3.59), the mean left lung (LL) score was5.8 (SD-2.37).

At the time of follow-up, different levels of abnormality in the lungs in the CT scans were still found in 49/51 patients; the mean radiological score was 10.9 (SD-8.87). The RL was affected more than the LL, with final mean scores of 6.2 (SD-5.46) and 4.7 (SD-3.67), respectively. The dominant location was the right lower lobe.

**Figure 1:**
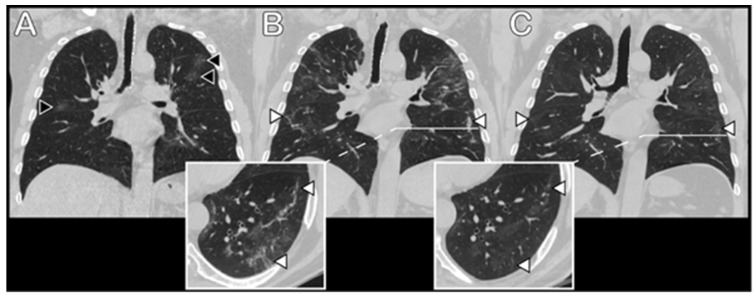
Coronal reconstructions and axial thin-section chest CT images of a 70-year-old man with confirmed COVID-19 pneumonia. (**A**) First CT scan 4 days after the onset of symptoms in the early stage of disease. Mild randomly distributed ground-glass opacities (black arrowheads) can be seen in both lungs. (**B**) CT scan at a later stage of disease (27 days after the onset of symptoms). Progression of previously observed ground-glass areas, as well as new areas of opacification can be seen. A reticular pattern emerged, with some parenchymal bands in peripheral parts of the lungs. (**C**) Follow-up CT scan 2 months later. A decrease in the density of ground-glass opacities can be seen, with only a slight decrease in the area of lesions. Some parenchymal bands persisted, with a decrease in abundance and thickness (white arrowheads).

**Figure 2:**
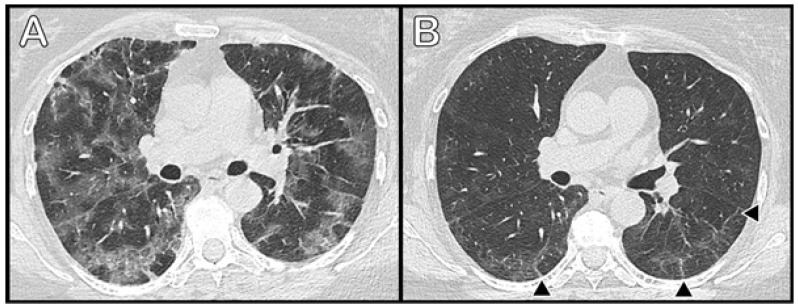
Axial thin-section chest CT images of a 60-year-old woman with confirmed COVID-19 pneumonia. (**A**) CT scan 10 days after the onset of symptoms at the peak stage of the disease. Multiple coalescing ground-glass opacities are seen in both lungs, with thickening of the interlobular septa in the lung periphery. (**B**) Follow up scan after 2 months. A decrease in density and size of ground-glass opacities can be seen, with persisting thickening of the interlobular septa and some parenchymal bands (black arrowheads) in peripheral parts of lungs.

Ground-glass opacity (GGO) was the most common radiological feature, found in 45/51 (88.2%) patients, with a mean score of 7.1 (SD-5.62); it was mostly observed in the RL (mean score of 4.0 (SD-2.6) and similarly in all lobes of the RL). Profibrotic radiological findings, with reticulation as the predominant feature, were found in 41/51 patients (80.4%). The right lung was also affected more than the left one (mean scores 4.1 (SD-3.39) and 2.9 (SD-2.40), respectively, with similar distribution in all lobes). Examples of radiological abnormalities are shown in Figure 1 and Figure 2.

We compared the radiological scores of groups with different disease severity. Statistically significant differences were found: the radiological scores were significantly higher in groups with severe and critical disease at the time of active disease as well as at the follow-up (*p* < 0.001).

When comparing the radiological scores over time between groups with different disease severity, statistically significant differences were found only in the group with moderate disease (*p* = 0.0019). When comparing values during active diseases and follow-up, the radiological scores for the groups with severe and critical disease were numerically lower at the follow-up (13.48 ± 3.89 vs. 11.48 ± 8.2 and 19.33 ± 4.05 vs. 18.5 ± 9.34), but the difference was not statistically significant (*p* = 0.2615 and *p* = 0.7476, respectively).

We also analyzed the correlation between radiological changes and impairment of DLCO. A statistically significant difference was found between the groups of patients with impaired and normal DLCO and the scoring of reticulation in the lungs (*p* = 0.022), but no significant difference was found when comparing these groups by the ground-glass opacity score (*p* = 0.877) or the total score (*p* = 0.254).

### 3.4. Health-Related Quality of Life

**Figure 3:**
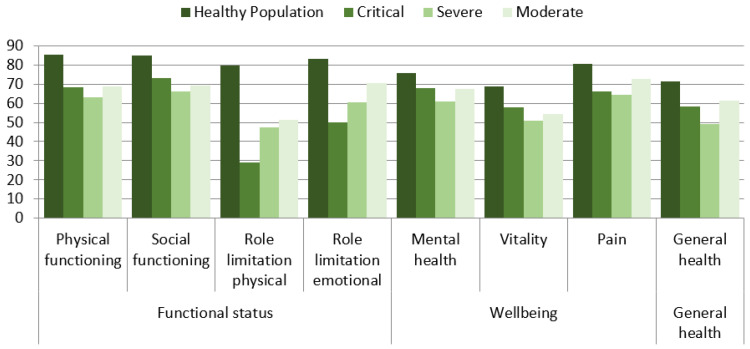
HRQoL results for groups with different disease severity and the general (healthy) population.

The SF-36 scores demonstrated a reduction in the reported health status across all domains in comparison with an age-matched population. The lowest scores were observed for limitations of social activities due to physical problems, vitality, and general health (Figure 3). No statistically significant difference between groups with different disease severity was found.

### 3.5. Laboratory Data

There were significant differences in all laboratory parameters depending on disease severity, except for leucocyte count (Table 3).

Laboratory parameters in patients with impaired and normal DLCO showed significant differences in the peak concentrations of CRP (*p* = 0.019) and IL-6 (*p* = 0.014) depending on disease severity.

## 4. Discussion

Our follow-up study showed that for the majority of the patients who survived moderate, severe, or critical COVID-19, two months are not sufficient for full regression of symptoms and recovery of HRQoL. Moreover, this period was not enough for half of the patients for the complete recovery of pulmonary function and physical capacity. For the final assessment of lung fibrosis or full morphological recovery, a longer follow-up is needed, as the different types and level of abnormality were still observed in more than two-thirds of the patients’ lungs 2 months after discharge from hospital.

Our analysis of the association between demographic characteristics and severity of COVID-19 complements most of the literature data. Male gender, obesity, and presence of comorbidities are highly associated with the severity of COVID-19 and a prolonged recovery period [20,21].

Almost all of our patients (48/51) still presented residual symptoms at the follow-up visit: fatigue, reduction of physical activity, dyspnea, and asthenia were the most common. These symptoms are similar to those published in the literature [22,23,24,25]. Though none of our patients had gastrointestinal impairment (GI), data about GI symptoms in the literature vary from none or rare [25,26] to up to presence in 20–30% of patients [23,27,28].

The data about COVID-19 impact on lung function and physical impairment at follow-up also varied. We found only one study (by A. Daher et al. [22]) showing no impairment in lung function 6 weeks after hospital discharge and no desaturation in the 6 MWT, though with a decreased 6 MWD. However, Brugger et al. showed [29] a DLCO reduction in 71.7% of patients. We observed impaired lung function at follow-up in 47.2% of the patients, with a predominant restrictive pattern and reduction of DLCO in one out of three (29.4%) cases. The same rate of DLCO impairment was found in the studies of Zhaoa et al. [27] and Lerum V. T et al. [30]. Moreover, similar data were observed in survivors of SARS and MERS [31]. Obstructive lung function abnormality is not characteristic of COVID-19, SARS, or MERS in the literature [22,27,30,31]. Our study data are in line with these results.

The variety of radiological findings in the lungs were still observed in the majority of our study cohort at follow-up. Ground-glass opacity (GGO) was the most common radiological feature, found in 88.2% of the patients. Other radiological pro-fibrotic findings, with reticulation as the predominant feature, were found in 80.4% of the patients. The results in the literature vary, with some studies reporting completely recovered lungs in 82% of patients [23] and others different levels of abnormality in up to 55% [32] or 80% [27,33] of the cases. These differences may be due to different levels of severity of COVID-19, presence of chronic lung disease prior SARS-CoV-2 infection, or a history of smoking. Moreover, the time of the follow-up in the studies also varied (established from symptoms’ onset, time of discharge, or 6, 8, or 12 weeks after discharge), and, as far as abnormalities in CT scans are prone to improve and/or change over time [28], the few weeks difference in the time of the follow-up visits might be significant for the results. High CT scores for inflammatory changes in follow-up studies, including ours, can be attributed to the tendency of ground-glass opacities to become less opaque, with only a minimal decrease in size, which is not taken into account when determining a score for the area of the lesion.

Our study results are similar to the data of Zhaoa et al. [27] and the data of SARS survivors 6 months after the disease [34].

M. Marvisi et al. [35] pointed out that 25% of COVID-19 survivors develop early fibrosis. Sine GGO was the predominant radiological feature in our study group, we suggest that the impairments in our cohort may improve and change over time. On the other hand, reticulation and other pro-fibrotic radiological changes were also found in 80.4% of patients; therefore, further follow-up CT scans are needed to evaluate the formation of fibrosis.

The HRQoL (SF-36) questionnaire scores in our study were reduced across all domains, with the lowest regarding limitations in social activities because of physical problems, vitality, and general health. Sar-van der Brugge et al. [29] showed similar results, with the exception of body pain. In this recent study, analysis of SF-36 scores and DLCO reduction showed a weak correlation that the authors interpretated as evidence of the fact that quality of life is determined by more aspects than physical functioning only. In our study, statistically significant differences were found between the groups with normal and reduced DLCO for limitations in social activities because of physical problems (*p* = 0.048), emotional status (*p* = 0.047), and general health (*p* = 0.007), suggesting that reduced lung function has a strong impact on quality of life, in combination with physical functioning and emotional status. Our results complement other publications data [36,37], showing lowered HRQoL scores at follow-up in multiple domains in COVID-19 survivors. Comparing SARS survivors, reduced HRQoL in multiple domains were observed even at 12 or 24 months [38,39]. Therefore, further follow-up is needed to evaluate this parameter.

There were significant differences in the majority of laboratory parameters between groups with different disease severity in our study cohort. Our results complement the data found in the literature [21]. These results and the significant differences found in the peak concentrations of CRP (*p* = 0.019) and IL-6 (0.014) between patients with impaired and normal DLCO suggest that lung damage is a complex process, determined not only by the direct effect of SARS-CoV-2, but also by immune system function and probable secondary bacterial infections in patients with severe and critical disease.

Our prospective follow-up study is one of very few studies performed in the European population that provides objective results for survivors of COVID-19 at different degrees of severity, without influence of former chronic lung damage. The main limitation of this study is the small number of patients and the limited follow-up period; more time is needed to supplement these data.

In conclusion, this research demonstrates that residual radiological and functional changes in the lungs, reduced physical activity, and HRQL status are found in a significant number of COVID-19 survivors 2 months after discharge from hospital. According to this data, a period of 2 months is not enough for patients’ complete recovery, and a longer follow-up is needed to determine the consequences of this disease.

## Figures and Tables

**Table 1:** Demographics and clinical characteristics of the patients: overall and comparison between disease severity groups.

			Total (*n* = 51)	COVID-19 Severity			*p*-Value
				Critical (*n* = 12)	Severe (*n* = 21)	Moderate (*n* = 18)	
Age years		Mean (SD) Min-Max	56.0 (11.7) 29–79	54.8(9.7)	60.0 (11.9)	52.1 (11.8)	0.104
Age group	18–30	*n* (%)	1 (2.0)	-	-	1 (5.6)	0.290
	31–40	*n* (%)	3 (5.9)	-	1 (4.8)	2 (11.1)	
	41–50	*n* (%)	13 (25.5)	5 (41.7)	3 (14.3)	5 (27.8)	
	51–60	*n* (%)	18 (35.3)	3 (25.0)	9 (42.9)	6 (33.3)	
	61–70	*n* (%)	10 (19.6)	4 (33.3)	3 (14.3)	3 (16.7)	
	71–80	*n* (%)	6 (11.8)	-	5 (23.8)	1 (5.6)	
Gender	Female	*n* (%)	26 (51.0)	1 (8.3)	11 (52.4)	14 (77.8)	<0.001
	Male	*n* (%)	25 (49.0)	11 (91.7)	10 (47.6)	4 (22.2)	
Medical history	Hypertension	*n* (%)	26 (51.0)	6 (50.0)	13 (61.9)	7 (38.9)	0.377
	Cardiac failure	*n* (%)	13 (25.5)	4 (33.3)	7 (33.3)	2 (11.1)	0.195
	Atrial fibrillation	*n* (%)	9 (17.6)	3 (25.0)	4 (19.0)	2 (11.1)	0.588
	Diabetes	*n* (%)	8 (15.7)	3 (25.0)	3 (14.3)	2 (11.1)	0.624
	Other diseases	*n* (%)	23 (45.1)	6 (50.0)	11 (52.4)	6 (33.3)	0.452
BMI groups	Normal weight	*n* (%)	9 (17.6)	1 (8.3)	2 (9.5)	6 (33.3)	0.018
	Overweight	*n* (%)	14 (27.5)	2 (16.7)	8 (38.1)	4 (22.2)	
	I° obesity	*n* (%)	15 (29.4)	6 (50.0)	7 (33.3)	2 (11.1)	
	II° obesity	*n* (%)	9 (17.6)	-	4 (19.0)	5 (27.8)	
	III° obesity	*n* (%)	4 (7.8)	3 (25.0)	-	1 (5.6)	

**Table 2:** Pulmonary function, physical capacity and radiological analysis at follow-up.

			Total (*n* = 51)	COVID-19 Severity			*p*-Value
				Critical (*n* = 12)	Severe (*n* = 21)	Moderate (*n* = 18)	
FVC	Normal	*n* (%)	47 (92.2)	8 (66.7)	21 (100.0)	18 (100.0)	0.002
	<LLN	*n* (%)	4 (7.8)	4 (33.3)	-	-	
FEV1	Normal	*n* (%)	46 (90.2)	8 (66.7)	20 (95.2)	18 (100.0)	0.012
	<LLN	*n* (%)	5 (9.8)	4 (33.3)	1 (4.8)	-	
FEV1/FVC	Normal	*n* (%)	50 (98.0)	12 (100.0)	21 (100.0)	17 (94.4)	0.588
	<LLN	*n* (%)	1 (2.0)	-	-	1 (5.6)	
TLC	Normal	*n* (%)	40 (78.4)	8 (66.7)	17 (81.0)	15 (83.3)	0.567
	<LLN	*n* (%)	11 (21.6)	4 (33.3)	4 (19.0)	3 (16.7)	
VC	Normal	*n* (%)	42 (82.4)	7 (58.3)	21 (100.0)	14 (77.8)	0.003
	<LLN	*n* (%)	9 (17.6)	5 (41.7)	-	4 (22.2)	
DLCO	Normal	*n* (%)	36 (70.6)	6 (50.0)	14 (66.7)	16 (88.9)	0.063
	<LLN	*n* (%)	15 (29.4)	6 (50.0)	7 (33.3)	2 (11.1)	
6MWD, m		Mean (SD)	553.5 (86.96)	518.8 (45.98)	550.7 (97.60)	575.4 (91.54)	0.258
6MWD, %		Mean (SD)	100.9 (14.97)	88.7 (14.30) *^,^**	102.6 (13.39) **	106.1 (13.50) *	0.008
Radiological score, acute disease, total		Mean (SD)	13.6 (5.77)	19.3 (4.05) *^,^**	13.48 (3.89) *	9.8 (5.58) **	<0.001
radiological score, follow-up, GGO		Mean (SD)	7.1 (5.62)	11.4 (6.64) *	7.3 (4.94)	3.8 (3.31) *	<0.001
radiological score, follow-up, fibrotic/reticular		Mean (SD)	3.9 (4.60)	7.1 (5.87) *	4.1 (4.49)	1.4 (1.58) *	0.002
radiological score, follow-up, total		Mean (SD)	10.9 (8.87)	18.5 (9.34) *^,^**	11.5 (8.21) *^,^***	5.22 (4.49) **^,^***	<0.001

6 MWD, 6 Minute Walking Distance, LLN, lower limit of normal. Radiological score, ground-glass opacification (GGO): in this score, all radiological inflammatory changes were included; radiological score, fibrotic/reticular: all radiological profibrotic radiological changes, such as reticulation, parenchymal bands, architectural distortion, bronchiectasis, were included. *, **, *** define pairwise difference calculated with Bonferroni post-hoc test.

**Table 3:** Laboratorial data and COVID-19 severity.

			Total (*n* = 51)	COVID-19 Severity			*p*-Value
				Critical (*n* = 12)	Severe (*n* = 21)	Moderate (*n* = 18)	
Max WBC, × 10^9^/L		Mean (SD)	8.63 (3.725)	9.60 (4.746)	9.49 (3.932)	6.98 (1.895)	0.063
Min LYM count, × 10^9^/L		Mean (SD)	1.00 (0.413)	0.74 (0.292) *	0.97 (0.347)	1.22 (0.455) *	0.006
CRP, mg/L		Mean (SD)	100.1 (97.97)	212.1 (122.08) *^,^**	84.3 (56.78) *	43.9 (47.72) **	<0.001
D-dimer, mcg/L		Mean (SD)	658.6 (689.47)	1302.5 (863.36) *^,^**	416.0 (225.05) *	512.3 (684.26) **	<0.001
IL-6, ng/L		Mean (SD)	71.9 (171.99)	189.0 (320.12) *^,^**	43.2 (42.86) *	18.4 (10.86) **	0.019
Ferritin, mcg/L		Mean (SD)	902.5 (992.28)	1823.0 (1113.63) *^,^**	895.7 (914.27) *	261.0 (212.31) **	<0.001
LDH, U/L		Mean (SD)	324.3 (106.09)	412.1 (91.88) *^,^**	331.6 (102.58) *^,^***	253.2 (65.52) **^,^***	<0.001
Hepatic injury	Yes	*n* (%)	38 (74.5)	12 (100.0)	17 (81.0)	9 (50.0)	0.005
	No	*n* (%)	13 (25.5)	-	4 (19.0)	9 (50.0)	
Renal injury	Yes	*n* (%)	18 (35.3)	11 (91.7)	6 (28.6)	1 (5.6)	<0.001
	No	*n* (%)	33 (64.7)	1 (8.3)	15 (71.4)	17 (94.4)	

Laboratorial parameters: peak WBC, min LYM concentrations, and peak CRP, D-dimer, IL-6, Ferritin, and LDH concentrations. Quantitative renal and hepatic injury was evaluated and compared. *, **, *** defines pairwise difference calculated with Bonferroni post-hoc test.

## Data Availability

Not applicable.
